# Examining the relationship between interpersonal support and retention in HIV care among HIV+ nursing mothers in Uganda

**DOI:** 10.1186/s13104-021-05639-z

**Published:** 2021-06-03

**Authors:** Jerry John Nutor, Pascal Agbadi, Thomas J. Hoffmann, Geoffrey Anguyo, Carol S. Camlin

**Affiliations:** 1 Department of Family Health Care Nursing, School of Nursing, University of California, San Francisco, 2 Koret Way, Suite N431G, San Francisco, CA USA; 2 Department of Nursing, Faculty of Allied Health Sciences, College of Health Sciences, Kwame Nkrumah University of Science and Technology, Kumasi, Ghana; 3 Department of Epidemiology and Biostatistics, and Office of Research, School of Nursing, University of California, San Francisco, San Francisco, CA USA; 4 Kigezi Healthcare Foundation, and Mbarara University of Science and Technology, Kabale, Uganda; 5 Department of Obstetrics, Gynecology and Reproductive Sciences, University of California, San Francisco, San Francisco, CA USA

**Keywords:** Breastfeeding, Lost to follow-up, New mothers, Interpersonal support, Sub-Saharan Africa

## Abstract

**Objective:**

The global burden of HIV on women and pediatric populations are severe in sub-Saharan Africa. Global child HIV infection rates have declined, but this rate remains quite high in sub-Saharan Africa due to Mother-to-child transmission (MTCT). To prevent MTCT of HIV, postpartum women living with HIV (WLHIV) are required to return to a health facility for HIV care within 60 days after childbirth (Retention in HIV care). Studies suggest that interpersonal support was positively associated with retention in HIV care. However, information on this association is lacking among postpartum WLHIV in Uganda. Therefore, this study investigates the relationship between interpersonal support, measured with the Interpersonal Support Evaluation List (ISEL-12), and retention in HIV care.

**Results:**

In a total of 155 postpartum WLHIV, 84% were retained in HIV care. ISEL-12 was negatively associated with retention in HIV care. Postpartum WLHIV retained in care (24.984 ± 4.549) have lower ISEL-12 scores compared to the non-retained group (27.520 ± 4.224), t(35.572) = − 2.714, p = 0.01. In the non-income earning sample, respondents retained in care (24.110 ± 4.974) have lower ISEL scores compared to the non-retained group (27.000 ± 4.855), t(20.504) = -2.019, p = 0.049. This was not significant among income earning WLHIV.

**Supplementary Information:**

The online version contains supplementary material available at 10.1186/s13104-021-05639-z.

## Introduction

Continuous engagement in HIV care is essential to prevent new infection, eliminate mother-to-child transmission (MTCT) and improve quality of life. In 2015, the World Health Organization (WHO) sets targets in an effort to expand access to antiretroviral therapy (ART) to people living with HIV. The policy, 90-90-90 (Treatment for All) target—90% of people with HIV diagnosed, 90% of all diagnosed people initiated on ART and 90% of those treated achieve viral suppression by 2020 which is followed by 95-95-95 to be achieved by 2030 [1]. However, in Sub-Saharan Africa (SSA) and South-East Asia (SEA), the majority of women living with HIV stop the ART after childbirth despite being available and free [2–4]. The reasons for stopping the ART are largely unknown, and a better understanding of the barriers to postpartum retention in HIV care is critical to reducing MTCT, improving population health and health equity, and maintaining the health of the mother and child, [5] which are goals for the WHO [6].

Uganda has one of the highest prevalence rates of HIV among pregnant women, with an estimated 120,000 pregnant women currently living with HIV [7] In 2017, 80% of HIV+ pregnant women in Uganda were enrolled in HIV care [6]. However, 6 months after childbirth, only 21% are retained in HIV care [8]. This situation requires further studies to ascertain the reasons for the inadequate retention. Previous studies have identified several barriers to the recommended retention in HIV care and optimal ART adherence during postpartum across the socioecological framework, from distal interpersonal and structural factors to individual-level determinants. Some of these barriers include lack of support, distance to a healthcare facility, HIV-related stigma, a poor patient-provider relationship and inadequate counselling [9–12]. Due to stigma and discrimination associated with HIV status, many WLHIV do not receive the needed interpersonal support [13]. Social support or interpersonal support is defined as the existence or availability of people who let an individual know that they care about, value, and love them [14]. Perceived social support contributes to retention in HIV care, ART adherence and general wellbeing of nursing mothers living with HIV [15, 16]. Postpartum period and HIV are two medically independent complex phenomena. When these phenomena are compounded by a lack of support, they can create significant challenges for the mother, child, and family. However, there is a paucity of studies on the relationship between interpersonal support and retention in HIV care among women particularly in the critical postpartum period in Uganda. Therefore, this study aimed to investigate the relationship between interpersonal support and retention in HIV care among nursing mothers in western Uganda. The study hypothesized that Nursing mothers living with HIV with increasing scores on an interpersonal support scale will have a higher likelihood to be retained in HIV care.

## Main text

### Methods

#### Study setting and design

This was a purposively sampled quantitative study conducted in five hospitals/health centers in the Kabale District of Uganda (KIHEFO Health Center, Rugarama Hospital, Rushhoroza Health Center, Kamukiira Health Centre and Kabale Regional Referral Hospital).

#### Recruitment and data collection

Midwives/nurses were the gatekeepers of the project and they were directly involved in purposively recruiting participants who meet the following condition: pregnant HIV+ women in their third trimester, enrolled in an ART program, and have understood the objective of the research and agreed to be part. The acceptance rate was 100%; all eligible women who were approached by the nurses/midwives agreed to participate in the study. After receiving consent from the participants, the midwives/nurses introduced the participants to trained research assistants who explained the study comprehensively to them and took their signed consent form and initial biographical details to enrol them in the study. The first phase of the recruitment occurred between June and August 2020. Three months after taking the details, the research assistants followed up on the consented participants to administer questionnaires that took data on their sociodemographic details and their commitment to returning to the facility for a check-up and take their antiretroviral drugs. The second phase of the data collection was conducted between October and December 2020. Questionnaires were administered to 167 respondents; a total of 155 (92.8%) of the respondents had near-complete data on the dependent and the independent variables and many of the control variables.

### Measures

### Dependent variable

A single question was asked to determine retention in HIV care. That is, “When was the last time you visited the hospital to collect your ARV drug or for a check-up?” Respondents who returned within 2 months (60 days) after delivery were considered to have retained in care [2, 17]. All other respondents who indicated that they have not returned or returned later than 2 months were considered non-retained in care.

#### Independent variable

The substantive independent variable was perceived social support measured with the Interpersonal Support Evaluation List-12 (ISEL-12) [18]. The ISEL-12 is a sum of twelve 4-point Likert scale questions that measure perceptions of social support by asking participants if they would be able to find assistance for various types of situations, with responses ranging from definitely false to definitely true (e.g. I feel there is no one I can share my most private worries and fears with) [18]. Scores range from 0 to 36, with higher scores indicating higher levels of perceived social support. An inter-item reliability test was performed, and the Cronbach alpha is an acceptable 0.7062 (Table 1). Detailed reports on the psychometric properties of ISEL-12 are reported elsewhere [18].

**Table 1:** Characteristics of the sample

Characteristics	n (%), unless indicated otherwise
Retention in HIV care	
No	25 (16.13)
Yes	130 (83.87)
ISEL [M(SD); min, max]	25.39^a^ (4.58); 9, 36
*Control variables*	
Age	
15–24 years	52 (33.55)
25–34 years	72 (46.45)
35 years+	31 (20.00)
Education	
None	13 (8.39)
Primary	110 (70.97)
Secondary	32 (20.65)
Cowife	
No	87 (58.00)
Yes	37 (24.67)
Don’t know	26 (17.33)
*Missing*	5
Number of children	
One child	38 (24.68)
Two children	37 (24.03)
Three children	41 (26.62)
Four children plus	38 (24.68)
*Missing*	1
Financial sufficiency	
Insufficient	128 (82.58)
Sufficient	27 (17.42)
Food insecurity	
Food insecure	74 (49.01)
Food secure	77 (50.99)
*Missing*	4
Monthly income	
No income	88 (56.77)
UGX 10,000+	67 (43.23)

^a^95% CI: 24.67, 26.12

#### Control variables

The following measures were included as control variables: age, polygynous marital status (measured by asking if the respondent was a wife/partner in a monogamous union or polygamous union), education, number of children, monthly income, self-reported financial sufficiency (measured as ‘sufficient’ or ‘not sufficient’), and food security. Household Food Insecurity Access Scale (HFIAS) [19] was used. The HFIAS is a sum of nine 4-point Likert scale questions, ranging from No (0), Rarely (1), Often (2), and (Always). An inter-item reliability test was performed, and the Cronbach alpha showed high reliability at 0.9433 (see Additional file 1). Detailed reports on the psychometric properties of HFIAS are reported elsewhere [19].

### Data analysis

Summary statistics such as percentages, mean, and standard deviations were used to describe the sample. Inter-item reliability tests were performed to determine the Cronbach Alpha of the two scales (HFIAS and ISEL-12) used in the study. A log-linear model implemented through a Poisson regression model with a robust variance estimate [20] was used to determine the association between the outcome and the main independent variable and the control variables in bivariate models. An additional file showing the bivariate risk ratios with their corresponding 95% confidence intervals between retention in HIV care and sample characteristics is available here (see Additional file 2). Two samples t-test with unequal variances was used to test the hypothesis of the study; these were done on the full sample and further stratified by income category. For the primary variable association of interest (perceived social support), results were considered significant if p < 0.05.

### Results

#### Sample characteristics

Sample characteristics are described in Table 1. Of the 155 HIV+ nursing mothers, 130 (84%) returned to a health facility for antiretroviral medication or general health check-up within 60 days after childbirth. The average interpersonal support score for the sample is 24.09 points (minimum point: 15, maximum point: 33). Women in the sample were relatively young (Mean = 28.13, SD = 6.285) and the majority (76%) were currently in union and living with their partner. The sample was socioeconomically diverse; 43% had income above UGX 10,000, 21% attained a secondary or higher education level, and 17% reported their financial situation as sufficient. About half of the respondents were in food-secure households (51%).

#### Correlates of retention in HIV care

There was a statistically significant relationship between ISEL-12 and retention in HIV care. None of the covariates was significantly associated with retention in HIV care (Table 2); therefore, the multivariable model building was not considered.

**Table 2:** Bivariate associations with retention in care

	Retention in HIV care		p-value
	No	Yes	
*Characteristics*	n (%), unless indicated otherwise		
ISEL [M(SD); min, max]	27.52^a^ (4.22); 20, 36	24.98^b^ (4.55); 9, 36	0.01
*Control variables*			
Age			0.520
15–24 years	10 (19.23)	42 (80.77)	
25–34 years	9 (12.50)	63 (87.50)	
35 years+	6 (19.35)	25 (80.65)	
Education			0.903
None	2 (15.38)	11 (84.62)	
Primary	17 (15.45)	93 (84.55)	
Secondary	6 (18.75)	26 (81.25)	
Cowife			0.267
No	11 (12.64)	76 (87.36)	
Yes	9 (24.32)	28 (75.68)	
Don’t know	4 (15.38)	22 (84.62)	
*Missing*			
Number of children			0.922
One child	5 (13.16)	33 (86.84)	
Two children	7 (18.92)	30 (81.08)	
Three children	7 (17.07)	34 (82.93)	
Four children plus	6 (15.79)	32 (84.21)	
*Missing*			
Financial sufficiency			0.435
Insufficient	22 (17.19)	106 (82.81)	
Sufficient	3 (11.11)	24 (88.89)	
Food insecurity			0.154
Food insecure	16 (20.78)	61 (79.22)	
Food secure	9 (12.16)	65 (87.84)	
*Missing*			
Monthly income			0.722
No income	15 (17.05)	73 (82.95)	
UGX 10,000+	10 (14.93)	57 (85.07)	

Two samples t-test with unequal variances was used to assess the association between ISEL-12 and Retention in HIV Care. Chi-square test of independence was used to assess the relationship between other covariates and Retention in HIV Care

^a^95% CI: 25.78, 29.26

^b^95% CI: 24.20, 25.77

#### The relationship between interpersonal support and retention in HIV care in Uganda

We examine the relationship between interpersonal support and retention in HIV care in Uganda using two samples t-test with unequal variances (Table 3). In the full sample, the results indicated interpersonal support was negatively associated with retention in HIV care among nursing mothers living with HIV (Table 3). Specifically, the full sample results indicated that nursing mothers living with HIV who are retained in care (24.984 ± 4.549) have significantly lower scores on ISEL-12 compared to the non-retained group (27.520 ± 4.224), t(35.572) = − 2.714, p = 0.01. In the stratified sample, the results further revealed that interpersonal support was negatively associated with retention in HIV care, although the association was only significant among the non-income earning respondents. Specifically, interpersonal support among non-income earning respondents who were retained in care (24.110 ± 4.974) was significantly lower compared to their counterparts in the non-retained group (27.000 ± 4.855), t(20.504) = − 2.019, p = 0.049 (Table 3).

**Table 3:** Interpersonal support and retention in HIV care stratified by income category

	Full sample		Non-income earning		Earned ≥ 10 k UGX	
	n	M (SD), [95% CI]	n	*M* (SD), [95% CI]	n	*M* (SD), [95% CI]
Retention in HIV care						
Retained	130	24.984 (4.549), [24.195, 25.774]	73	24.110 (4.974), [22.949, 25.270]	57	26.432 (3.673), [25.127, 27.084]
Non-retained	25	27.520 (4.224), [25.776, 29.263]	15	27.000 (4.855), [24.311, 29.689]	10	28.300 (3.129), [26.061, 30.538]
Difference		− 2.535		− 2.890		− 2.194
t statistic		− 2.714		− 2.091		− 1.989
p-value		0.010		0.049		0.067
Satterthwaite's df		35.572		20.504		13.789

First, given the results, we, therefore, failed to accept the hypothesis that nursing mothers living with HIV with increasing scores on an interpersonal support scale will be more likely to be retained in HIV care. Further, personal income differences were observed in the association between ISEL-12 and retention in HIV care.

### Discussion

About 16% of the respondents were not retained in care. We unexpectedly found that interpersonal support was negatively associated with retention in HIV care in Western Uganda. There are important implications to these findings. First, the nature of interpersonal support received by the nursing mothers living with HIV may be unrelated to the drive needed to motivate nursing mothers living with HIV to visit the health facility for a medical check-up or antiretroviral treatment.

Also, lack of disclosure of HIV status to social relations due to fear of stigmatization and discrimination, which is often the case, can limit the nursing mothers living with HIV to interpersonal supports that have no direct bearing on motivation to be committed to antiretroviral treatment. Contrary to our findings, several studies have reported the positive effect of social support on retention in HIV care [21–24]. Given the unique deviation of our study from the reported influence of social support on retention in HIV care in the literature, [21, 22, 24] further studies using diverse social support measuring scales can provide further clarity on this relationship in Western Uganda.

Furthermore, in a stratified analysis, we found that interpersonal support was negatively associated with retention in HIV care among women who were not earning income, but this relationship was not significant for income-earning nursing mothers living with HIV. As revealed in the results, the lack of positive effect of social support on retention in HIV care is more severe among the non-income earning nursing mothers living with HIV than their counterparts who earned some monthly income.

For the income-earning HIV+ nursing mothers, given the non-significant influence of interpersonal support on retention in HIV care, their income might be their greatest asset in maintaining them in care since they can afford to pay for the transportation cost associated with visits to the health facilities for the antiretroviral treatment [9, 10, 25, 26].

### Conclusion

To conclude, our study unexpectedly suggests that interpersonal support is negatively associated with retention in HIV care among nursing mothers living with HIV. The findings also suggest the need for attending nurses and midwives to conduct a general assessment of women during the antenatal period to understand their available support to appropriately plan for their care after discharge home from the hospital following childbirth. Future studies should use longitudinal and mixed methods to investigate the impact of retention in HIV care and loss to follow up on the health of the women and their babies.

## Limitations

Our study had a few limitations. First, the sample size was small to draw very valid conclusions and hence we recommend that future studies use a large sample size. Secondly, it was not possible in this setting to obtain a probability-based sample; thus, the results cannot be generalized for the nursing mothers living with HIV in western Uganda. Thirdly, as a cross-sectional study, the associations observed in this study do not infer a causal relationship between the independent and outcome variables. Finally, it is important to note that the data presented in this study were self-reported; therefore, the ratings are prone to recall and social desirability bias.

## Supplementary Information

**Additional file 1.** An additional file showing Reliability analysis results of the ISEL-12 and the HFIAS scale.

**Additional file 2.** An additional file showing the bivariate risk ratios with their corresponding 95% confidence intervals between retention in HIV care and sample characteristics.

## Data Availability

All data supporting the results and conclusion of this paper are included in the article.

## Abbreviations

ART: Antiretroviral therapy
CI: Confidence intervals
HIV: Human Immuno-deficiency Virus
HFIAS: Household Food Insecurity Access Scale
ISEL: Interpersonal Support Evaluation List
MTCT: Mother-to-child transmission
WHO: World Health Organization
WLHIV: Women Living with Human Immuno-deficiency Virus
