# Supplementary material for: Examining the relationship between interpersonal support and retention in HIV care among HIV+ nursing mothers in Uganda
Source: BMC Res Notes. 2021 Jun 3;14:224. doi: 10.1186/s13104-021-05639-z (PMC8176692; doi:10.1186/s13104-021-05639-z)
Supplement: Supplementary file 1 — Additional file 1. An additional file showing Reliability analysis results of the ISEL-12 and the HFIAS scale. [file 13104_2021_5639_MOESM1_ESM.docx]

**Additional file 1: Reliability analysis results of the ISEL-12 and the HFIAS scale**

| ISEL: Interpersonal Support Evaluation List | | | HFIAS: Household Food Insecurity Access Scale | | |
| --- | --- | --- | --- | --- | --- |
| Items | Mean (SD) | α | Items | Mean (SD) | α |
| **ISEL-12** |  | **0.7062** | **HFIAS** |  | **0.9433** |
| ISEL 1 | 1.826 (0.975) | 0.6791 | HFIAS 1 | 0.669 (0.833) | 0.9400 |
| ISEL 2 | 1.774 (1.023) | 0.6794 | HFIAS 2 | 0.804 (0.918) | 0.9365 |
| ISEL 3 | 2.432 (0.603) | 0.6781 | HFIAS 3 | 0.763 (0.897) | 0.9339 |
| ISEL 4 | 2.348 (0.599) | 0.6752 | HFIAS 4 | 0.753 (0.881) | 0.9348 |
| ISEL 5 | 2.174 (0.722) | 0.6956 | HFIAS 5 | 0.614 (0.859) | 0.9327 |
| ISEL 6 | 2.284 (0.788) | 0.7069 | HFIAS 6 | 0.619 (0.885) | 0.9344 |
| ISEL 7 | 2.213 (0.756) | 0.6729 | HFIAS 7 | 0.432 (0.739) | 0.9356 |
| ISEL 8 | 1.877 (0.863) | 0.6972 | HFIAS 8 | 0.396 (0.699) | 0.9383 |
| ISEL 9 | 2.335 (0.677) | 0.6971 | HFIAS 9 | 0.286 (0.625) | 0.9432 |
| ISEL 10 | 2.381 (0.595) | 0.6860 |  |  |  |
| ISEL 11 | 1.968 (1.968) | 0.6874 |  |  |  |
| ISEL 12 | 1.781 (0.942) | 0.6965 |  |  |  |
| ISEL 1. If I wanted to go on a trip for a day (for example to the beach, the country or mountains), I would have a hard time finding someone to go with me.  ISEL 2. I feel that there is no one I can share my most private worries and fears with.  ISEL 3. If I were sick, I could easily find someone to help me with my daily chores.  ISEL 4. There is someone I can turn to for advice about handling problems with my family.  ISEL 5. If I decide one afternoon that I would like to go to a movie that evening, I could easily find someone to go with me.  ISEL 6. When I need suggestions on how to deal with a personal problem, I know someone I can turn to.  ISEL 7. I don’t often get invited to do things with others.  ISEL 8. If I had to go out of town for a few weeks, it would be difficult to find someone who would look after my house or apartment (the plants, pets, garden, etc.).  ISEL 9. If I wanted to have lunch with someone, I could easily find someone to join me.  ISEL 10. If I was stranded 10 miles from home, there is someone I could call who could come and get me.  ISEL 11. If a family crisis arose, it would be difficult to find someone who could give me good advice about how to handle it.  ISEL 12. If I needed some help in moving to a new house or apartment, I would have a hard time finding someone to help me. | | | HFIAS 1. In the past four weeks, did you worry that your household would not have enough food? If yes, how often did this happen?  HFIAS 2. In the past four weeks, were you or any household member not able to eat the kinds of foods you preferred because of a lack of resources? If yes, how often did this happen?  HFIAS 3. In the past four weeks, did you or any household member have to eat a limited variety of foods due to a lack of resources? If yes, how often did this happen?  HFIAS 4. In the past four weeks, did you or any household member have to eat some foods that you really did not want to eat because of a lack of resources to obtain other types of food? If yes, how often did this happen?  HFIAS 5. In the past four weeks, did you or any household member have to eat a smaller meal than you felt you needed because there was not enough food? If yes, how often did this happen?  HFIAS 6. In the past four weeks, did you or any other household member have to eat fewer meals in a day because there was not enough food? If yes, how often did this happen?  HFIAS 7. In the past four weeks, was there ever no food to eat of any kind in your household because of lack of resources to get food? If yes, how often did this happen?  HFIAS 8. In the past four weeks, did you or any household member go to sleep at night hungry because there was not enough food? If yes, how often did this happen?  HFIAS 9. In the past four weeks, did you or any household member go a whole day and night without eating anything because there was not enough food? If yes, how often did this happen? | | |
