# Supplementary material for: Examining the relationship between interpersonal support and retention in HIV care among HIV+ nursing mothers in Uganda
Source: BMC Res Notes. 2021 Jun 3;14:224. doi: 10.1186/s13104-021-05639-z (PMC8176692; doi:10.1186/s13104-021-05639-z)
Supplement: Supplementary file 2 — Additional file 2. An additional file showing the bivariate risk ratios with their corresponding 95% confidence intervals between retention in HIV care and sample characteristics. [file 13104_2021_5639_MOESM2_ESM.docx]

**Additional file 2: Characteristics of the Sample and Bivariate Associations with Retention in Care**

|  | **Overall Sample**  **(n=155)** | **No income**  **(n=88)** | **Monthly income of UGx 10000+**  **(n=67)** |
| --- | --- | --- | --- |
|  | **Crude** | **Crude** | **Crude** |
|  | RR [95% CI] | RR [95% CI] | RR [95% CI] |
| ***Independent variable*** |  |  |  |
| ISEL* | 0.981*  [0.966,0.996] | 0.980*  [0.961, 0.999] | 0.976  [0.951,1.002] |
| ***Control variable*** |  |  |  |
| **Age** |  |  |  |
| 15-24 years | 1 | 1 | 1 |
| 25-34 years | 1.083  [0.924,1.270] | 1.072  [0.864, 1.330] | 1.094  [0.860,1.392] |
| 35years+ | 0.998  [0.803,1.242] | 0.992  [0.749, 1.313] | 1.011  [0.712,1.434] |
| **Education** |  |  |  |
| None | 1 | 1 | 1 |
| Primary | 0.999  [0.781,1.278] | 0.931  [0.699, 1.239] | 1.125  [0.715,1.771] |
| Secondary | 0.960  [0.721,1.279] | 1.029  [0.735, 1.439] | 0.966  [0.588,1.588] |
| **Cowife** |  |  |  |
| No | 1 | 1 | 1 |
| Yes | 0.866  [0.709,1.058] | 0.892  [0.682, 1.658] | 0.836  [0.616,1.133] |
| Don’t know | 0.969  [0.807,1.163] | 1.003  [0.785, 1.282] | 0.929  [0.704,1.224] |
| **Number of children** |  |  |  |
| One child | 1 | 1 | 1 |
| Two children | 0.934  [0.765,1.140] | 0.816  [0.619, 1.076] | 1.059  [0.812,1.381] |
| Three children | 0.955  [0.792,1.151] | 0.848  [0.662, 1.086] | 1.068  [0.826,1.382] |
| Four children plus | 0.970  [0.805,1.168] | 0.918  [0.749, 1.126] | 0.968  [0.672,1.395] |
| **Financial sufficiency** |  |  |  |
| Insufficient | 1 | 1 | 1 |
| Sufficient | 1.073  [0.919,1.254] | 0.798  [0.355, 1.795] | 1.126  [0.933,1.360] |
| **Food insecurity** |  |  |  |
| Food insecure | 1 | 1 | 1 |
| Food secure | 0.902  [0.782,1.040] | 0.811  [0.638, 1.029] | 0.985  [0.793,1.223] |
| **Monthly Income** |  |  |  |
| No income | 1 |  |  |
| UGX 10,000+ | 1.026  [0.893,1.178] |  |  |
| Exponentiated coefficients; 95% confidence intervals in brackets; *p<0.05 | | | |
